# Pemphigus—The Crux of Clinics, Research, and Treatment during the COVID-19 Pandemic

**DOI:** 10.3390/biomedicines9111555

**Published:** 2021-10-28

**Authors:** Branka Marinović, Joško Miše, Ines Lakoš Jukić, Zrinka Bukvić Mokos

**Affiliations:** 1 Department of Dermatology and Venereology, University Hospital Centre Zagreb, School of Medicine University of Zagreb, Šalata 4, 10000 Zagreb, Croatia; branka.marinovic@kbc-zagreb.hr (B.M.); ilakosju@gmail.com (I.L.J.); 2 Department of Dermatology and Venereology, University Hospital Centre Zagreb, European Reference Network (ERN)-Skin Reference Centre, Kišpatićeva 12, 10000 Zagreb, Croatia; joskojerolim@gmail.com

**Keywords:** pemphigus, desmoglein, rituximab, immunodermatology, COVID-19, SARS-CoV-2

## Abstract

Pemphigus is a rare autoimmune disease characterised by the production of pathogenic autoantibodies in response to different desmosome proteins. The pathophysiological process leads to the development of blisters and erosions on mucosal and/or skin surfaces. The classical clinical variants of pemphigus are pemphigus vulgaris and pemphigus foliaceus. A diagnostic delay is very common in pemphigus, especially among patients with mucosal involvement. However, in recent years we have witnessed considerably fewer patients with extensive mucocutaneous manifestations, since patients with oral lesions are referred to dermatologists to start the treatment much sooner than they had been previously. Among non-classical variants of pemphigus, unusual cases with discrepancies between autoantibody profiles and clinics challenge the “desmoglein compensation theory”. The identification of several other autoantigens that perform a role in the pathogenesis of different variants of pemphigus will progress immunodermatology towards an approach that will determine personalized pemphigus subtypes for each patient. Comorbidities among patients are primarily associated with the prolonged use of corticosteroids and other immunosuppressive agents. The SARS-CoV-2 pandemic raised concerns regarding the immunosuppressive effects of treatment and the risk of a more complicated COVID-19 infection, as well as on the ability to develop an adequate vaccine response.

## 1. Introduction

Pemphigus diseases are a group of rare autoimmune bullous diseases that affect the skin and mucous membranes. They are immunopathologically characterised by the production of pathogenic autoantibodies that are directed against different proteins of desmosomes, leading to acantholysis and the formation of vesicles, blisters, and erosions on the skin and/or mucous membranes. Desmoglein 1 (Dsg 1) and desmoglein 3 (Dsg 3) are the primary target antigens in pemphigus. They belong to the cadherin gene family of Ca^2+^ dependent transmembrane adhesion molecules, which are found within and outside of desmosomes—adherence structures connecting neighbouring keratinocytes. In addition to producing antibodies against Dsg 1 and Dsg 3, several other antibodies against molecules such as desmocollin (Dsc), muscarinic and nicotinic acetylcholine receptors, pemphaxin, mitochondrial proteins, and thyroid peroxidase have been detected in pemphigus [1].

The disease is chronic and relapsing in nature, with a life-threatening and devastating impact on the patients’ quality of life. Pemphigus is a rare disorder with an incidence of around 3.7 new patients per 1 million inhabitants per year in Croatia [2]. Worldwide, the incidence rate is reported to be between 0.76 to 16.1/1,000,000 inhabitants, with the highest incidence in the Jewish population. The disease usually manifests between the ages of 45 and 65 years, with a female predominance, between 1:1.1 and 1:1.7 female/male ratio in various populations [3]. The golden standard for the diagnostics of pemphigus is a biopsy of an intact vesicle for histopathology and perilesional skin for direct immunofluorescence microscopy (DIF). The result of a histopathology presents acantholysis with suprabasal blistering as a symptom of pemphigus vulgaris and subcorneal blistering in pemphigus foliaceus, whereas the DIF finding is characterised by an intercellular IgG/C3 deposition in the epidermis with a fluorescence pattern resembling a honeycomb [4]. Indirect immunofluorescence (IIF) and enzyme-linked immunosorbent assays (ELISA) detect circulating autoantibodies against desmogleins. ELISA provides information regarding the target antigen in pemphigus, distinguishing among various pemphigus variants [4].

Different types of pemphigus have been identified based on the clinical and histopathological features, as well as on the specific antigens against which the autoantibodies are produced. The primary forms are pemphigus vulgaris (PV) and pemphigus foliaceus (PF). Furthermore, in the last decades, other forms of pemphigus, also known as “non-classical forms”, have been observed and described: pemphigus herpetiformis, IgA pemphigus, pemphigus seborrheicus, pemphigus erythematosus, paraneoplastic pemphigus, and drug-induced pemphigus. PV is the most common clinical form of pemphigus, accounting for approximately 70% of cases; it is also considered the most severe form of the disease [4].

## 2. Clinical Presentation

### 2.1. Pemphigus Vulgaris

The clinical manifestation of PV may present mucosal or mucocutaneous involvement. Nearly all patients develop mucosal lesions, primarily in the oral mucosa, with or without cutaneous lesions.

Oral lesions provide the first manifestation in 50–70% of cases and occur in 90% of patients during the course of the disease [5]. The blisters are rarely intact because they break rapidly, and patients present with painful oral lesions, with the most affected areas being the buccal and palatine mucosa, lips, and gingiva. In addition, lip lesions often present with hemorrhagic crusts. Other mucous membranes may be affected too, though rarely, including nasal mucosa, conjunctivae, pharynx, larynx, oesophagus, and genital mucosa.

The skin involvement presents with flaccid blisters of a clear content on normal or erythematous skin. The blisters are fragile and break easily, developing in painful bleeding erosions covered by crusts. Predilection sites are the scalp, face, and intertriginous areas and areas of mechanical irritation such as armpits, buttocks, shoulders, and elbows, but any site of the body covered with stratified squamous epithelium can be affected; the palms and soles usually remain unaffected [5].

Studies have proven that the clinical manifestation of PV is defined by the Dsg autoantibody profile, with mucosal PV presenting reactivity against Dsg3 and mucocutaneous PV against Dsg 1 and Dsg 3 [6]. Furthermore, Dsg 3 antibodies are ineffective in causing cutaneous-only lesions due to the co-expressed Dsg 1 [7]. In addition, the severity of mucosal lesions was positively correlated with an increase in Dsg 3 levels, while the severity of cutaneous lesions was positively correlated with the level of Dsg 1 autoantibodies [8]. This concept is known as “desmoglein compensation theory”, which has been established as a textbook explanation of pathogenesis and clinical manifestation of its pemphigus subtypes [9]. However, it is worth noting that although there is a correlation between the clinical phenotype and antibody profile, the former cannot be regarded in absolute terms as there are cases where discrepancies exist, such as mucosal PV with Dsg1 antibodies, mucocutaneous PV without Dsg 3 antibodies, and cutaneous PV lacking Dsg3 antibodies [10]. Furthermore, recent studies have confirmed the existence of autoantibodies targeted at other non-Dsg1 and 3 antigens in pemphigus patients, providing the explanation for discrepancies and the evidence for the refutation of “desmoglein compensation theory”. The additional antigens that play a role in the cell-to-cell adhesion are: desmosomal antigens (Dsg 2 and Dsg 4, Dsc 1–3 and desmoplakins 1 and 2); collagen XVII; cell-membrane receptors, such as nicotinic acetylcholine receptor subunits a3 and a9; pemphaxin (also called annexin 31); FceRIa and thyroperoxidase [11].

There may be differences in the onset of pemphigus symptoms. In the majority of cases, patients firstly present with mucosal lesions. Thereafter, with an average lag period of 4 months, they proceed to develop cutaneous lesions [12]. This clinical manifestation is due to the difference in timing of the presence of antibodies against Dsg 1 and Dsg 3. Several studies have shown that Dsg 3 antibodies were identified in 90–98% of all patients at the time of the initial diagnosis, while positive Dsg 1 antibodies were detected in only 63% of patients with PV at the time of diagnosis [13]. The clinical significance of this pathophysiological process in PV development explains the diagnostic delay: since around 60–80% of patients experience oral lesions first, and the disease remains limited to mucosal surfaces in 1 in 4 patients, diagnostic delays are commonly observed in PV [14]. According to the study conducted in 2000 by Sirois, 80% of patients first developed oral lesions with a diagnostic delay greater than six months. The study from 2020, that was conducted in Turkey on 36 newly diagnosed patients, revealed that 20 years later, the diagnostic delay has not shortened and remains firmly at 6.2 months, with all of the patients who presented oral mucosa involvement were initially misdiagnosed [15]. However, the knowledge and research on PV have greatly improved in the recent decades, along with the awareness that both early diagnosis and treatment are crucial to prevent fatal complications of the disease. This leads to the hypothesis that improvements in PV diagnostics should have shortened the diagnostic delay, as we have observed in the day-to-day work at our Department. In the past decades, we have seen more patients with extensive mucocutaneous lesions affecting a large area of skin, whereas in recent years, owing to better diagnostic procedures and collaboration between dermatologists and oral pathologists, we are witnessing evidently fewer patients with pemphigus manifestations of both mucosa and skin. We have observed that patients who develop erosions in their oral mucosa, that do not heal, are referred to dermatologists much sooner than they would have been 10 or 20 years ago. They are diagnosed and begin the treatment earlier and do not develop extensive cutaneous lesions as we have witnessed before. However, further research over a longer period of time is needed to support this observation.

The advent of rituximab has also altered the classical mucocutaneous clinical presentation of PV. The increasing evidence for the successful use of rituximab proved to be a breakthrough in the treatment of pemphigus in the last two decades. Rituximab, a monoclonal antibody directed against the CD20 antigen on B-lymphocytes, depletes CD20 B cells from circulation and has been used in B-cell lymphoma, rheumatoid arthritis, and off-label autoimmune dermatologic conditions [16]. In a randomised controlled trial from 2017, Joly et al. demonstrated that 89% of patients with PV and PF assigned to the rituximab group achieved complete remission through therapy compared to 34% of patients who were assigned to the treatment with prednisone alone [17]. Since then, rituximab has been licensed for the treatment of moderate to severe pemphigus in the United States and the European Union. More recently, the European Academy of Dermatology and Venereology (EADV) and an international panel of experts recommended intravenous CD20 inhibitors (rituximab) as a first-line therapy option for moderate to severe pemphigus [18,19]. Table 1 summarises the treatment strategies. The results have revolutionised the management of PV as there are many studies published in recent years suggesting that rituximab is an effective agent in inducing remission when used as the first-line treatment; the reported rituximab efficiency in achieving complete remission ranges from 58% to over 90% of the patients included in the observed studies [20,21,22,23,24,25]. Even though relapses generally occur at 6–24 months after the first treatment, with each subsequent rituximab cycle, a substantially longer remission period is achieved [26]. This new frontier in the PV treatment, along with better diagnostic approaches, has decreased the frequency of patients with progressed mucocutaneous disease that require lengthy hospitalisations, prolonged corticosteroid therapy and its side effects.

### 2.2. Pemphigus Vegetans

Pemphigus vegetans is a rare form of pemphigus vulgaris, accounting for 1–2% of all cases of pemphigus [27]. It is clinically characterised by the formation of vesicles, bullae, pustules, and erosions that form vegetating plaques with excessive granulation tissue and crusts, especially in the intertriginous areas, face, and scalp. There are two clinical subtypes of pemphigus vegetans: the Neumann type, which is considered severe, beginning with vesicles and blisters that rupture forming hypertrophic erosions and exudative vegetating masses; and the Hallopeau type, which is regarded as benign and begins with pustules that rupture and form vegetating erosions [28]. A typical clinical sign described in pemphigus vegetans is the extensive involvement of the tongue, known as the cerebriform tongue [29].

### 2.3. Pemphigus Foliaceus

PF is a pemphigus variant where the mucosal surfaces are intact. This is due to the presence of Dsg 1 and the absence of Dsg 3 antibodies [30]. The blister formation occurs considerably high in the subcorneal region of the epidermis, making the blisters very fragile, even more so than those in PV. Intact blisters may not be seen at all. Patients present with erosions and scaly or crusty erythematous patches. In the scalp, fissured crusts are usually apparent [4]. Although pemphigus foliaceus is often reported in the literature as a milder form of pemphigus with a better prognosis, according to our experience, those patients tend to develop erythroderma, and in this form, PF is quite resistant to different forms of therapies, including newer therapies.

## 3. Non classical Pemphigus Clinical Variants

Since the 1970s, other types of pemphigus have been described with clinical and immunopathological features that separate them from the classical variants of pemphigus. Accurate diagnosis of these forms of pemphigus is essential as the appropriate treatment may differ from the conventional pemphigus variants.

### 3.1. Pemphigus Herpetiformis (PH)

PH is a rare form of pemphigus, accounting for less than 10% of all cases. It is characterised by clinical manifestations that resemble dermatitis herpetiformis and histological findings that, although widely heterogeneous, are consistent with pemphigus [31]. Patients present with atypical clinical features that are not usually present in PF and PV such as grouped vesicles, blisters, erosions, and crusts on erythematous skin in a herpetiform composition with a frequently associated pruritus. IIF and ELISA detect IgG antibodies against Dsg 1 and less commonly against Dsg 3, Dsc 1 and 3, and an unknown 178-kDa protein [32]. PH usually runs a benign course and responds well to treatment, even with low doses of corticosteroids. The combination therapy of systemic steroids with dapsone has presented the most promising results, with most patients achieving complete remission [33].

### 3.2. IgA Pemphigus

IgA pemphigus is a very rare autoimmune vesiculopustular disease clinically characterised by flaccid bullae or erosions on the skin. There are two types of IgA pemphigus: subcorneal pustular dermatosis (SPD) and intraepidermal neutrophilic IgA dermatosis (IEN). Patients present with vesicles or pustules on the erythematous plaques. SPD typically presents with “half-half blisters” where the bottom section contains yellow non-infectious pus, and the top section contains clear fluid [34]. The IEN-type presents deeper atypical pustules often forming a “sunflower-like” configuration [35]. The predilection sites are the trunk and proximal parts of the extremities with intertriginous areas, such as the axillary and groin regions, being the most commonly affected. The autoantigen of SPD-type is Dsc 1, but that of the IEN-type is yet to be confirmed, although some cases have suggested the production of IgA antibodies for either Dsg 1 or Dsg 3 [36]. The clinical presentation and course of the disease are milder and more benign than classic pemphigus [35]. Systemic corticosteroids are the mainstay of therapy, with reports and evidence of dapsone, isotretinoin, acitretin, mycophenolate mofetil, and adalimumab inducing remission in treating IgA pemphigus [35,37].

### 3.3. Paraneoplastic Pemphigus

Paraneoplastic pemphigus (PNP) is a rare pemphigus entity that manifests as polymorphic mucocutaneous eruptions in a patient with an underlying neoplasm. It is characterised by the production of autoantibodies against various target antigens, mainly plakin family proteins (most common envoplakin and periplakin) [38]. In approximately two-thirds of the cases, the skin disease occurs in patients with an existing neoplasm, and in the remaining one-third of cases, neoplasms are detected after the mucocutaneous disease occurs. The most observed clinical characteristic of PNP is stomatitis, which is the earliest symptom of the disease and is highly resistant to therapy [39]. Stomatitis presents with painful erosions and ulcerations of the oropharynx extending to the vermilion borders of the lip. Most patients also suffer from severe conjunctivitis. Anogenital lesions have also been observed. In some patients, PNP only presents with mucosal involvement. The cutaneous lesions of PNP are quite varied, with a mixture of blisters, erosions, and target lesions that mimic those of PV, PF, or bullous pemphigoid. Another typical clinical feature of PNP is lichenoid eruptions, which are similar to that in lichen planus or the lichenoid type of chronic graft-vs-host disease [38]. The most severe extracutaneous manifestation is bronchiolitis obliterans, which is the leading cause of death in these patients. Four features that are often referred to as the minimal criteria for PNP diagnosis, have been generally accepted: (1) clinical features of severe stomatitis with or without polymorphic cutaneous eruptions, (2) histologic features of acantholysis and/or interface dermatitis, (3) the demonstration of anti-plakin autoantibodies and (4) the presence of an underlying neoplasm [38]. Haematologic malignancies are the most frequent underlying neoplasms associated with PNP. Non-Hodgkin lymphoma is the most frequent neoplasm, followed by Castleman’s disease and chronic lymphocytic leukemia. The non-haematologic neoplasms associated with PNP include thymoma (malignant and benign), sarcoma, malignant melanoma, and bronchogenic squamous cell carcinoma [40]. The most commonly used treatment for PNP includes systemic corticosteroids in combination with other immunosuppressive agents such as cyclosporine, azathioprine, and mycophenolate mofetil. Rituximab has also been used, achieving a positive response, but results are less consistent than in PV [41,42]. Complete remission is rarely achieved, considering the pathogenic role of both humoral and cellular immunity in PNP. The prognosis of PNP is poor, with a 5-year survival rate at only 35–40% [43].

### 3.4. IgG/IgA Pemphigus

IgG/IgA pemphigus is characterised by the clinical and histological features of pemphigus mediated by IgG and IgA antibodies. Over thirty years ago, the first reported cases of an intercellular pattern of IgG and IgA antibodies were found in the DIF [44]. Clinical presentations reveal a heterogeneity with the characteristics of IgA pemphigus, pemphigus vulgaris, pemphigus foliaceus, and, less frequently, paraneoplastic pemphigus, and pemphigus vegetans. Most patients develop cutaneous lesions with vesicles, bullae, and pustules present in an annular morphology. Although mucosal involvement was not considered to be typical for IgG/IgA pemphigus, some recent studies revealed that 40% of patients experienced mucosal involvement [45]. Since some studies report almost one-third of IgG/IgA cases as having an underlying malignancy, patients with this type of pemphigus require particular attention [46].

There has been a debate as to whether IgG/IgA pemphigus exists as a single distinct entity or a transitional phase along a spectrum of IgG pemphigus to IgA pemphigus. Studies have confirmed the role of IgG and of IgA to Dsg 1 and Dsg 3 as the predominant antibodies among these patients, but several studies have reported Dsc 1 and Dsc 3 as target antigens too [47]. One explanation for this heterogeneity, which is shown to be a characteristic of IgG/IgA pemphigus, is the epitope spreading phenomenon in which an inflammatory event releases new target antigens, exposes them to the immune system, and then induces subsequent autoimmunity to new related antigens [48]. However, it is thought that a class switching of antibodies does not occur with epitope spreading as the limiting of the occurrence of epitope spreading to only class-switched cells is a mechanism by which the body limits the damage experienced as a result of the autoimmune disease [49]. Another explanation of the IgG/IgA heterogeneity is the existence of IgA antibodies at the onset of IgG pemphigus, however, at below the threshold for detection by DIF. A study by Mentink et al. from 2007 showed that of 100 patients with IgG Dsg 1 and/or Dsg 3 antibodies, 54 were also found to have IgA anti-desmoglein antibody levels [50].

The practical importance of understanding these pathophysiological mechanisms is apparent when we are faced with patients who do not respond as expected to conventional pemphigus therapy. We suggest re-evaluating the case of these patients for antibody profiles that are absent in the initial diagnosis. Dapsone has been recommended as the first-line therapeutic option for IgG/IgA pemphigus. Combination therapy with systemic steroids is also an option [43].

Further research is needed to better understand the pathogenesis, potential therapeutic implications, and the relationship of IgG/IgA pemphigus with malignancies.

### 3.5. Drug-Induced Pemphigus

Many of the known triggering factors have been linked to pemphigus, but drugs continue to be one of the most prevalent potential causes of the disease. The medications associated with the triggering of pemphigus can be classified into three groups: thiol drugs, phenol drugs, and non-thiol/phenol drugs [51]. Among thiol groups, most cases of drug-induced pemphigus have been linked with the use of penicillamine, lisinopril, and bucillamine. They are the most often reported drugs that are related to pemphigus induction [52]. Phenol drugs include aspirin, rifampicin, and levodopa. A number of other non-thiol and non-phenol drugs that have been associated with pemphigus are non-steroid anti-inflammatory drugs and calcium channel blockers. In addition, there have been sporadic reports of other medications being linked to the pathogenesis of pemphigus, such as biologics (secukinumab and tocilizumab) [53].

The mechanism by which medications trigger pemphigus is biochemical and immunological. Drugs with a thiol group have the ability to activate proteolytic enzymes, interfere with the cell-to-cell adhesion of the keratinocytes, and to bind Dsg 1 and Dsg 3. The phenol group causes acantholysis by participating in the regulation and synthesis of complement and proteases [52]. A systematic review of 170 patients presenting with drug-induced pemphigus published in 2021 revealed that pemphigus vulgaris (38.9%), pemphigus foliaceus (33.5%), and paraneoplastic pemphigus (3.6%) were the most common subtypes in patients presenting with cutaneous (68.6%), mucocutaneous (30.1%) and mucosal (1.3%) involvement [54].

The first step in the management of drug-induced pemphigus is the discontinuation of the inciting medication. In the majority of cases, further treatment was needed with systemic corticosteroids and immunosuppressive agents such as azathioprine, methotrexate, and mycophenolate mofetil. The prognosis of drug-induced pemphigus is good, and treatment outcomes are better than in classic pemphigus cases. Almost 90% of patients achieve long-lasting clinical remission [54]. However, drug-induced pemphigus is a diagnostic challenge; since histopathological and clinical features are identical to idiopathic pemphigus, a thoroughly documented patient history is crucial to timely and adequately identify the causative drug.

### 3.6. “Unusual” Pemphigus Manifestations

In the past decade, studies have been conducted regarding unusual pemphigus cases that challenge the “desmoglein compensation theory” as there is evidence that antibody specificities and titers do not always correlate to the clinical features and disease activity of pemphigus. There are reports in the literature regarding patients with anti-Dsg3 positivity in the absence of oral mucosal involvement, patients with oral mucosal involvement despite anti-Dsg3 negativity, cutaneous pemphigus vulgaris cases lacking Dsg1 autoantibodies, and the discordance between clinical activity and Dsg titers [55]. Some studies suggest that the discrepancy between clinical presentation and serology ranges between 36% and 48% of all cases [11].

The frequency of patients presenting with unusual clinical and serological phenotypes is difficult to estimate. Some studies report a high number of cases with discrepancies, however, we have observed only one such patient at our Department in the past decade. A 78-year-old female patient developed facial cutaneous lesions without mucosal involvement with positive IgG antibodies to Dsg 3 and negative anti-Dsg 1 antibodies, confirmed by immunoblotting and ELISA [56].

The reality may be that different patients develop distinct sets of antibodies to various antigens. Considering that recent findings suggest that 30% to 50% of patients with clinical presentations of pemphigus challenge “desmoglein compensation theory”, we ourselves expected more patients with unusual pemphigus manifestations [11]. However, in practical terms, the availability of biomarkers that would allow us to personalise each patient’s pemphigus subtype remains difficult to realise. In many clinical settings, the resources to develop and standardise such molecular diagnostics are scarce, both in terms of human and financial capabilities. Nonetheless, in the future, with the advancement in diagnostics and its affordability, immunodermatology will transition towards an approach that will identify personalised pemphigus subtypes for each patient.

## 4. Pemphigus and Comorbidities

The mortality rate associated with pemphigus diseases has decreased significantly since the 1950s—from approximately 75% to 20% in the last two decades [57]. The use of systemic corticosteroids as the primary method of treatment has led to the increased survival of patients, albeit not without its cost. The prolonged use of corticosteroids and other immunosuppressive medications has led to the development of numerous treatment-related comorbidities among pemphigus patients (Table 2). The well-known and harmful side effects of corticosteroids, such as Cushing’s syndrome, osteoporosis, cataracts, glaucoma, and adrenal suppression, have been strongly associated with pemphigus [58]. The importance of these iatrogenic side effects is even greater because it has been proven that patients with a secondary diagnosis of pemphigus have a significantly higher level of mortality than those patients admitted to the hospital for pemphigus [58]. These findings suggest that the comorbidities related to pemphigus are more responsible for the number of deaths than the diagnosis of pemphigus.

The advent of rituximab has revolutionised the treatment methods for pemphigus. The most significant achievement of rituximab is not only the efficacy in inducing clinical remission but also in reducing the need for corticosteroid therapy, thereby allowing for rapid corticosteroid tapering. Several studies demonstrated that patients treated with rituximab had significantly less corticosteroid exposure and were less likely to experience severe or life-threatening corticosteroid-related side effects [60]. However, treatment with an immunosuppressive agent, such as rituximab, requires caution. Recent studies report that almost all patients receiving rituximab underwent infusion-related events (hypertension or hypotension), and between 4.5% and 10% of patients experienced severe adverse events, all of infectious aetiology (perirectal phlegmon, meningitis, B streptococcal infection leading to septic shock, urinary tract infection, *Pneumocystis jirovecii* pneumonia) [23,25].

The connection between pemphigus and several other autoimmune and inflammatory conditions has been described in the literature. Several cross-sectional and observational studies have demonstrated the association of pemphigus with rheumatoid arthritis, diabetes, myasthenia gravis, autoimmune thyroid diseases, systemic lupus erythematosus, alopecia areata, ulcerative colitis, and multiple sclerosis [61,62,63]. There is a higher prevalence of hidradenitis suppurativa among patients with pemphigus, with those requiring prolonged therapy at an increased risk of developing a more severe form of hidradenitis suppurativa [64]. The association between pemphigus and psoriasis has long been established. The increased incidence of hidradenitis suppurativa and psoriasis among patients with pemphigus proves the occurrence of a complex immunological interplay between cutaneous autoimmune and autoinflammatory conditions. Furthermore, patients with pemphigus experience an increased risk for malignancies compared to the general population. Several studies conducted in the United States, Germany, and Israel have shown an association between haematological malignancies, gastrointestinal and oropharyngeal neoplasms and PV and PF [58,65,66].

Further investigation is required to better characterise the association of autoimmune and inflammatory conditions and malignancies in patients with pemphigus. The improved survival chances of pemphigus patients and their prolonged exposure to systemic immunosuppressive treatments requires an improved access to dermatological care and a multidisciplinary approach in screening for potential comorbidities.

## 5. Pemphigus and COVID-19

Since the beginning of the COVID-19 outbreak, several concerns have been raised by dermatologists and pemphigus patients who take immunosuppressive drugs. Rituximab irreversibly affects humoral immunity, and the reconstitution of B-cell immunity may require several months, which can cause severe problems for patients who contract SARS-CoV-2. There have been cases reported of a more complicated SARS-CoV-2 infection in patients with autoimmune bullous diseases (AIBD) who have taken rituximab during the last year [67]. However, the cessation of the first-line treatment option for pemphigus can lead to the exacerbation of the disease and to life-threatening complications, which require lengthy hospitalisations, considered to be a risk during the COVID-19 outbreak. Considering the recommendation to minimise both the level and the duration of immunosuppressive therapy during the COVID-19 pandemic, some authors suggested the use of low-dose rituximab protocol (two infusions of 500 mg rituximab, two weeks apart) in patients with mild-to-moderate pemphigus [68]. Dermatologists should approach each patient individually to ensure proper disease control with minimal immune suppression to avoid any severe exacerbations and potentially fatal outcomes.

Over recent months, concerns have been raised regarding the effect of rituximab on the SARS-CoV-2 vaccine response. It has been suggested that patients receiving rituximab may have a weaker immunological response to the vaccine which may persist for 6 to 12 months after rituximab infusion [69]. Recently, several studies on the SARS-CoV-2 vaccine response, for both mRNA and viral vector, among patients with an immune-mediated inflammatory disease have been published [70]. Among the several immunosuppressive therapies, these studies found the most significant reduction in the immune response of patients receiving B-cell depletion therapy, most notably rituximab [71,72,73]. The timing of immunization is of crucial importance, as some authors provide evidence of an attenuated yet meaningful vaccine response six months after dosing, whereas other case series have observed that patients receiving rituximab failed to develop a sufficient antibody response even six months after their last dose [74,75]. These conflicting results should not discourage clinicians from recommending the vaccination to their patients with AIBD who are receiving rituximab, as vaccine-induced immunity has both a humoral and a cell-mediated response. The same study that found an impaired humoral response to rituximab showed that all patients developed SARS-CoV-2 specific T-cell reactivity, identified through an interferon-gamma response to SARS-CoV-2 peptides [75]. By considering all of these perspectives into account, there is a consensus regarding the timing of the vaccination and rituximab therapy, that the vaccine should be administered at least four weeks before the first rituximab infusion or 12 to 20 weeks after completing a treatment cycle to allow for the sufficient immune response to develop [76]. Since the vaccine response is slower in patients with AIBD receiving rituximab, they should be reminded to seriously adhere to the guidelines of at least two weeks after the final dose to consider themselves fully vaccinated and, nonetheless, to follow epidemiological measures of masking and social distancing after the two weeks. The option of receiving a third (“booster”) dose, once available according to the national guidelines on SARS-CoV-2 vaccination, should be encouraged for patients.

Since the first outbreak of the COVID-19 pandemic (in March of 2020), we have faced several challenges regarding the treatment of pemphigus patients. During the first few months of the pandemic, healthcare systems worldwide were required to focus on the care of patients with COVID-19—which was, at the time, a new disease that still had to be understood. In addition, older patients and those with chronic diseases were advised to postpone hospital visits whenever was possible. This particularly affected immunosuppressed patients, including those with pemphigus. Furthermore, a lack of understanding regarding the new SARS-CoV-2 virus infection led to inconsistent expert recommendations concerning immunomodulatory and immunosuppressive therapy for pemphigus [77,78,79]. Consequently, we were encouraged to use teledermatology resources to closely monitor patients on corticosteroid and other immunosuppressive therapy, whereas the use of rituximab was limited. The use of teledermatology platforms was well received by the patients, thereby suggesting it to be a valuable tool in day-to-day dermatology practice. Furthermore, we tapered the immunosuppressive therapy on maintenance doses where possible and provided the necessary information on adherence to health principles, social distancing, and vaccination. However, new cases and patients with severe exacerbations were advised to refer to our outpatient department and day hospital. The exacerbation of pemphigus in two of our patients who had to stop or postpone rituximab during the first wave of COVID-19 in the spring of 2020 had to be managed via an increased corticosteroid dosage in day hospital. Table 3 summarises the approach strategies to patients with pemphigus during the COVID-19 pandemic, with regards to management, therapy and the risk of COVID-19.

The experience of the COVID-19 pandemic resulted in the availability of new data regarding AIBD patients. The systematic review of 732 AIBD patients receiving immunomodulatory therapies during the COVID-19 pandemic revealed that these patients did not display higher rates of SARS-CoV-2 infection or more severe symptoms of COVID-19 than the general population [82]. In contrast, other authors reported that patients with AIBDs or rheumatic diseases who acquired and died of COVID-19 were more likely to receive rituximab and that the risk of contracting COVID-19 decreased with each month after receiving rituximab [83,84,85]. Therefore, current international expert recommendations for the management of AIBDs during the COVID-19 pandemic highlight the necessity of the individualised approach when deciding on the initiation of rituximab, and the use of rituximab as the maintenance therapy is not recommended [86].

In our experience, the availability of the SARS-CoV-2 vaccine presented the pivotal moment with regards to the rituximab treatment of pemphigus patients [87]. Currently, we use rituximab in severe and/or conventional therapy-resistant cases, following recommendations regarding SARS-CoV-2 vaccination [76]. We have also played a role in the post-COVID-19 outpatient unit with several patients developing suspected autoimmune cutaneous post-COVID-19 manifestations. Whether the COVID-19 pandemic increased the diagnostic delay of pemphigus or impacted the incidence of pemphigus by other means remains to be seen.

## 6. Conclusions

Although the scientific knowledge concerning the pathophysiology of the pemphigus group of diseases is progressing, and despite new therapeutic modalities, primarily rituximab, there are still many questions that need to be resolved. The identification of a complete autoantibody profile, and not only antidesmosomal, in each patient with pemphigus will advance an understanding of the diversities among patients.

## Figures and Tables

**Table 1:** Treatment of PV.

	1st Line Treatment	No Disease Control at Week 3–4
Mild PV [18] (involved BSA < 5% and limited oral lesions not impairing food intake or requiring analgesics; PDAI score ≤ 15	Rituximab (two infusions of 1 g two weeks apart) alone or associated with prednisone 0.5 mg/kg/d Prednisone 0.5–1.0 mg/kg/d with or without azathioprine (2.0 mg/kg/d) or mycophenolate mofetil 2 g/d or mycophenolate sodium 1440 mg/d	Initially treated with rituximab and prednisone: - increase the prednisone up to 1.0 mg/kg/d or - add intravenous corticosteroids pulses Initially treated with prednisone alone: - add rituximab (2 × 1 g)
Moderate to severe PV [18] (involved ≥ 15% BSA, multiple mucosal involvement, severe oral lesions or dysphagia with weight loss, significant pain; moderate PV PDAI score > 15 and ≤45; severe PV PDAI score > 45)	Rituximab (two infusions of 1 g two weeks apart, associated with prednisone 1 mg/kg/d Prednisone 1–1.5 mg/kg/d alone or with an immunosuppressive drug (azathioprine 1 to 2.5 mg/kg/d) or mycophenolate mofetil 2 g/d or mycophenolate sodium 1440 mg/d)	Initially treated with rituximab and prednisone: - increase the prednisone up to 1.5 mg/kg/d or - add intravenous corticosteroids pulses Initially treated with prednisone alone: - increase the prednisone dose to 1.5 mg/kg/d PLUS add rituximab (2 × 1 g) or - add immunosuppressive drug (azathioprine 1 to 2.5 mg/kg/d or mycophenolate mofetil 2 g/d or mycophenolate sodium 1440 mg/d).

BSA: body surface area; PDAI: pemphigus disease area index; PV: pemphigus vulgaris.

**Table 2:** Major side effects of systemic glucocorticoids [59].

Dermatologic	Thin skin
	Purpura
	Ecchymoses
	Acne
	Increased hair growth (hirsutism)
	Facial erythema
	Striae
	Cushingoid appearance
Ophthalmologic	Cataract
	Glaucoma
Cardiovascular	Fluid retention
	Hypertension
	Arteriosclerosis
	Arrhythmias
Bone and muscle	Osteoporosis
	Avascular necrosis
	Proximal myopathy
Neuropsychiatric	Euphoria
	Emotional disturbances
	Depression
	Insomnia
	Pseudotumor cerebri
Metabolic	Hyperglycemia
	Hypokalemia
Endocrine	Supression of hypothalamic-pituitary-adrenal axis
	Obesity (truncal)
Immune system	Immunosuppression
Gastrointestinal	Gastritis
	Peptic ulcer disease
	Steatohepatitis

**Table 3:** Pemphigus during the COVID-19 pandemic.

	Clinics	Management	Therapy	Risk of COVID-19
Mild-to-moderate (PDAI ≤ 45)	Risk of exacerbation due to discontinuation of treatment	Outpatient unit, day-hospital	Tapering corticosteroids	Higher if corticosteroid dose > 20 mg and if other immunosuppressive agents administered [80]
Severe (PDAI > 45)	Risk of exacerbation due to discontinuation of treatment; risk of more severe form of COVID-19 due to high doses of immunosuppressive agents	Hospitalization with negative PCR test	RTX on a case to case basis depending on local infection rate, underlying comorbidities, adherence to epidemiological measures, and full vaccination [81]	Higher if RTX administered [80]

PDAI: Pemphigus Disease Area Index; RTX: rituximab.

## Data Availability

Not applicable.
